# A conditional embrace—Swedish LGBTQ+ spaces through the eyes of ethnic minority non-heterosexual individuals

**DOI:** 10.3389/fpsyg.2022.1009192

**Published:** 2022-12-16

**Authors:** Emelie Louise Miller

**Affiliations:** ^1^Department of Psychology, Mid Sweden University, Sundsvall, Sweden; ^2^Department of Psychology and Social Work, Faculty of Human Sciences, Mid Sweden University, Östersund, Sweden

**Keywords:** LGBTQ+, ethnicity, intersectionality, minority stress, separatism, Sweden, ideals, resilience

## Abstract

**Introduction:**

In research on sexuality, marginalized sub-groups within sexual minorities have often been overlooked. From the vantage point of Sweden, internationally ranked as an exemplary progressive nation in equality issues and LGBTQ+ rights, and with an increasingly diversified population, the lived experiences of ethnic minority non-heterosexual people are still very much lacking in voice and visibility. The present study aimed to examine experiences within Swedish non-heterosexual spaces, held by ethnic minority non-heterosexual individuals.

**Method:**

A thematic analysis of in-depth interviews with 22 Swedish non-heterosexual individuals, 13 cis-men and nine cis-women, with diverse first- and second-generation immigration backgrounds, was conducted.

**Results:**

Two main themes were identified. The first theme, “*Constantly contested identities*,” is composed of the sub-themes “*Ingrained, intersecting ideals*” and “*Prejudiced spaces*,” and the second theme, “*Effects and counteractions*,” of the sub-themes “*Never fully human*” and “*Representation and separatism*.” The results, presented starting from a more theoretical level, moving to situated knowledge, and finally to psychological and practical implications, demonstrate that ethnic minority non-heterosexual people experience problematic and intersecting ideals, with related discrimination, in various Swedish non-heterosexual settings. Experiences of alienation, exotification, and tokenism were common among the participants and had negative psychological effects, including multiple-minority stress and a constant outsider feeling. Representation and participation in separatist forums were utilized as primary strategies to counteract the negative effects.

**Discussion:**

The findings shed light on previously under-researched ideals and actions within Swedish LGBTQ+ spaces, and raises questions about how positive belonging can be achieved for multiple-minorities. Further research and continued critical discussions about ethnic minority non-heterosexual people's plight within non-heterosexual settings in Sweden, and beyond, is advocated.

## Introduction

The present study concerns the human longing for belonging and under-researched obstacles to achieving this. The study focuses on ethnic minority non-heterosexual individuals within a supposedly open-minded context: Swedish non-heterosexual spaces. From the existing international literature, it is clear that the LGBTQ+ world is both a place for the celebration of diversity but at the same time, nonetheless, mirrors mainstream society and as such is steeped in discriminatory attitudes and actions, including racism, misogyny, and ageism (Phua and Kaufman, 2003; Heaphy et al., 2004; Wood, 2004; Han, 2007; Ward, 2008; Miller, 2015, 2019; Robinson, 2016; Grollman, 2018; Siverskog et al., 2019). In the “Western” non-heterosexual world, gay has often been equaled with “white,” directly or indirectly prompting a whiteness ideal, which exposes ethnic minority non-heterosexual individuals to racial discrimination and expectations of adaption (Han, 2007; Velez et al., 2015; Cyrus, 2017; Ghabrial, 2017; Patel, 2019). Other ideals, within non-heterosexual settings, include the anti-effeminacy ideals that repeatedly have marginalized “feminine” non-heterosexual men who don't conform to masculine norms (Taywaditep, 2002; Clarkson, 2006; Eguchi, 2009; Brennan et al., 2013; Nash, 2013; Hunt et al., 2016; Savenije, 2016; Murgo et al., 2017; Salvati et al., 2021). Negative emotions toward “feminine” gay men, as well as experiences of femme-negativity and femme-related stigma, have also been found among non-heterosexual women (Blair and Hoskin, 2016; Salvati et al., 2018; Hoskin, 2019). Racism, misogyny, and other discriminatory practices have dire consequences for those who are discriminated, including minority stress (e.g., Velez et al., 2015; Ghabrial, 2019; Schmitz et al., 2019). Sweden is internationally ranked as an exemplary progressive nation in equality issues and LGBTQ+ rights, offering a unique perspective on multiple marginalizations (Flores et al., 2018; Lagerberg, 2018; World Economic Forum, 2018; ILGA—The International Lesbian, 2022; IPSOS, 2022). Globally, there still exists a noticeable scarcity of intersectional research exploring ethnic minority non-heterosexual people's experiences, mainly because sexuality and ethnicity historically have been researched separately (Bowleg et al., 2003; Collins, 2004; Clarke et al., 2013; Almeida and Rolim Neto, 2020). From a Swedish perspective, this literature shortage is even more pronounced. In the present study, the intersection of ethnicity and sexuality is in focus.

### Demographics and discrimination pertaining to ethnicity and sexuality

Collecting and defining data on ethnicity is an arduous task, owing to the ever-changing and multi-faceted nature of ethnic identification and nation-based differences in categorization and available demographics (Office for National Statistics, 2022). Racism is likewise a difficult-to-navigate concept, with a myriad of interpretations (Hylland Eriksen, 2019). In North America, which frequently has constituted an unofficial departing point in international comparisons, racial categorization such as White/Caucasian, Black, Asian, and Hispanic is often applied in, for example, college admissions (Michel et al., 2019). This is very different from the Swedish “practice,” where such categorization is not officially used. A discourse of “colorblindness,” which indirectly states that race is a non-issue in Sweden, has been promoted, hindering constructive discussions about racism (Dovemark, 2013; Hübinette, 2014). While “race” is seldom used, “foreign background” is an accepted and applied terminology, and the available data indicate that 34% of Sweden's total population have a foreign background (see Table 1). For the foreign-born, the most common countries of origin are Syria, followed by Iraq, Finland, Poland, Iran, Somalia, Afghanistan, and Yugoslavia (Statistics Sweden, 2022b). Returning to North America, racial discrimination is a pervasive problem, with over 50% of Black, Hispanic, and Asian people reporting racial discrimination (Lee et al., 2019). Similar data are available in, e.g., Brazil, where significant differences are found in racial victimization between Black and White Brazilians (Truzzi et al., 2022). Even if such documentation of racial discrimination can't readily be compared with Swedish conditions, racism clearly exists in both overt and covert forms in Sweden (Akrami et al., 2000; Hylland Eriksen, 2019; Quillian et al., 2019; Kristoffersson et al., 2021). Violent actions by the successor groups to *Vitt ariskt motstånd* (VAM, “White Aryan Resistance”) still occur, as does everyday racism (Integrationsverket, 2002; Hällgren, 2005; DO Diskrimineringsombudsmannen, 2014; Hylland Eriksen, 2019; Kristoffersson et al., 2021). In 2015, in the wake of the worsening situation in Syria, 163,000 refugees applied for asylum in Sweden, a figure double the one in 2014 (Statistics Sweden, 2022b). The dominant public and political discourse moved from massive initial support to a discourse of the refugee situation being unsustainable and provoking (Dahlgren, 2016; Lindholm, 2020; Wernesjö, 2020). The nationalistic political party “Sweden Democrats” is as of now the second largest party in Sweden (Valmyndigheten, 2022). Police-reported hate crimes are the closest to “racial categorization” found in Sweden, with 28% of the most recent documented crimes categorized as Afrophobic hate crimes, 3% as anti-gypsy hate crimes, 1% as hate crimes against the Sami; however, the remaining 68% were categorized as “other” xenophobic and racist hate crimes (Brottsförebyggande rådet, 2022). In line with international statistics, for instance, from the UK (Office for National Statistics, 2022), greater ethnic diversity is found in Sweden's big cities, and the proportion of foreign-born individuals varies greatly depending on the geographical region. In Östersund, a town in the Northern part of Sweden, 10% of the inhabitants are foreign-born, compared to 43% in Botkyrka (a part of Stockholm; Statistics Sweden, 2022c). Independent of how long you have lived in Sweden, looking non-European, having a “non-Swedish” name, or talking with a “non-Swedish” accent is likely to result in various forms of discrimination and a decreased chance of positive belonging (Hällgren, 2005; Lindholm, 2020).

**Table 1:** Existing population data on the ethnic and sexual minority make-up of Sweden.

**Ethnicity, *N*** ** (year 2021)**	**Sexual orientation, *N*** **(based on new marriages^a^ in 2021)**
Foreign born: 2,090,503	Different-sex new marriages: 70,355
Swedish-born with two foreign-born parents: 662,069	Same-sex new marriages, women: 607
Swedish-born with one foreign-born parent: 805,340	Same-sex new marriages, men: 356
Swedish- born with two Swedish-born parents: 6,894,414	Total number of new marriages: 71,291
Total population: 10,452,326	Total number of same-sex marriages: 936 = *1,4% of total number of marriages*
Residents with foreign background: 3,557,912 = *34% of total population*	

a In cases where the partner is not registered in Sweden and information on gender is missing, the assumption has been made that it is a different-sex couple. The statistics are based on the persons' legal gender; i.e., other markers of gender identities are not accounted for Statistics Sweden (2022a,b).

Source: Statistics Sweden (2022a,b).

Similar to demographics on ethnicity, no official data exist on sexual orientation—percentage and/or identification—in Sweden (Statistics Sweden, 2022c). An indication of the non-heterosexual proportion can be found in a survey of 27 countries, where 12% of Swedes stated that they were only or mostly attracted to the same sex (IPSOS, 2022). Official statistics related to sexual orientation are limited to data on married couples (see Table 1). Globally, societal acceptance of non-heterosexuality remains distinctly divided by country, but numbers and nuances are, again, not reliably mapped out (Pew Research Center, 2020). Many countries still criminalize and penalize non-heterosexuality (Pew Research Center, 2020; Younes, 2020). It is estimated that 83% of sexual minorities globally conceal their sexual orientation, and the proportion is calculated to be region-dependent, with, e.g., 95% of sexual minorities in the Middle East and North African regions concealing their sexual minority orientation compared to 37% in Northern/Western European regions (Pachankis and Bränström, 2019). A large proportion of adults worldwide are unable or unwilling to define their sexual orientation, even in anonymous surveys (IPSOS, 2022). Differences in the acceptance of sexual minorities are affected by the level of education, religion, political ideology, and national wealth (Pew Research Center, 2020). In Sweden, with a GDP per capita of over $50,000, acceptance of non-heterosexuality is among the highest in the world. By contrast, in Nigeria and Ukraine, with GDP < $10,000, acceptance is among the lowest (ibid.). Internationally, Sweden is known as a defender of LGBTQ+ rights (Jungar and Peltonen, 2015; Carlson-Rainer, 2017; Jungar and Peltonen, 2017; Flores et al., 2018; ILGA—The International Lesbian, 2022). This positive reputation appears to be substantiated. Sweden for example ranked highest concerning acceptance (79%) of same-sex marriages^1^, and Swedes demonstrate the highest (71%) support for LGBTQ+ anti-discrimination laws (IPSOS, 2022). In most parts of the world, including Sweden, younger people are more accepting of sexual minorities compared to their older counterparts (Pew Research Center, 2020). Stigma and discrimination based on sexuality are however not extinct in Sweden, and non-heterosexual Swedes still demonstrate lower wellbeing compared to heterosexual Swedes (Björkenstam et al., 2016; Bränström, 2017; Clark et al., 2021).

### Intricate intersections

Through an intersectional lens, researchers attempt to understand both the meaning and consequences of multiple identity categories and how these intersect and inform lived experiences (e.g., Crenshaw, 1991; Cole, 2009). An intersectional perspective is needed to grasp how oppression and privilege are created by intersecting and varying hierarchies of gender, ethnicity, class, and sexuality, among others (Purdie-Vaughns and Eibach, 2008; Fitzgerald, 2017). The latest concluding remarks from the UN Committee on the Elimination of Racial Discrimination (CERD) criticized the lack of information on intersecting forms of discrimination in Sweden (UN Committee on the Elimination of Racial Discrimination, 2018). In a systematic literature review of 68 international scientific papers, upholding both a sexual and an ethnic minority position resulted in increased marginalization and lack of support (Duran, 2019). A scarcity of literature on the intersections of sexuality and ethnicity exists in Sweden. For instance, a search in ProQuest Social Sciences conducted in June 2022 with the below search string yielded 11 scholarly journal results in total (excluding duplicates): (ab) “Sexual minorit^*^” OR non-heterosexual^*^ OR homosexual^*^ OR LGBT^*^ OR gay OR lesbian OR same-sex OR queer AND (ab) “ethnic minorit^*^” OR immigrant^*^ OR refugee^*^ OR racialized OR “people of color” AND (ab) Sweden. Using the same search words in the same database on the same date, but removing the intersection, Sweden + sexual minorities yielded roughly 300 results, and Sweden + ethnic minorities yielded roughly 2,000 results. When widening the search to global findings, i.e., the same searches in the same database but with no country limit, 177,886 results on sexual minorities, 297,494 results on ethnic minorities, and 5,616 results on the intersection of these two were found. In other words, 13% of existing sexual and ethnic minority studies (without the intersection) in Sweden focus on sexual minorities, while the proportion internationally is higher, with 37% focusing on sexual minorities. Looking at the ratio of intersectional vs. “one-minority” focus on these topics, it was observed that only 1% of the international search results emphasize this particular multiple-minority group. In Sweden, the number is even lower, with barely 0.5% of the search results highlighting the intersecting identities. While there exists a sizeable body of international literature on ethnic minorities and sexual minorities, there is a relative lack of research on the intersection of ethnicity and sexual orientation and this dis-representation has not seen significant progress over the last two decades (Ghabrial and Ross, 2018; Kavanaugh et al., 2020; Clark et al., 2021). Among the relevant “intersectional” Swedish search results, two studies demonstrated how far-right populist parties, including the Sweden Democrats, portray themselves as champions of LGBTQ+ rights and pose “backward immigrants” as the main threat to these rights, a logic based on ideas of European superiority (Spierings and Zaslove, 2015; Duina and Carson, 2020). Another large population-based study reported that Swedish non-heterosexual people demonstrate substantially elevated odds of all mental health outcomes compared to heterosexual people (Clark et al., 2021). The same study advocated for intersectionality-focused research to gain a better understanding of unique factors associated with wellbeing among non-heterosexual people with immigrant backgrounds in Sweden (Clark et al., 2021).

### Intersectional minority stress and resilience

Minority stress theory highlights the relevance of minority identities in the stress process and is based on the premise that prejudice and stigma directed toward a minority group involve unique stressors that can result in adverse health outcomes (Meyer, 2003; Lick et al., 2013; Meyer and Frost, 2013). As an example, mental health disparities between heterosexual Swedes and non-heterosexual Swedes can largely be explained by minority stress theory (Bränström, 2017; Clark et al., 2021). Intersectional minority stress theory aims at explaining processes that create health inequalities among multiple minorities (Velez et al., 2015; Cyrus, 2017; Schmitz et al., 2019). The international literature indicates that sexual stereotypes are embedded in racial stereotypes and that racism and sexism intersect in ways that negatively affect ethnic minority non-heterosexual individuals' coming out processes, mental health, and body image (Nagel, 2003; Brennan et al., 2013; Brown, 2014; Ikizler and Szymanski, 2014; Velez et al., 2017; Robinson and Frost, 2018). Both interpersonal and structural discrimination contribute to multiple-minority stress among ethnic minority non-heterosexual people (e.g., Calabrese et al., 2015; Ching et al., 2018). Ethnic minority non-heterosexual individuals often experience tensions between their ethnic and sexual orientation identities, sometimes to the extent that they are perceived as mutually exclusive (Galarza, 2013; Sarno et al., 2015; Lim and Hewitt, 2018; Kehl, 2019). Light-skinned ethnic minority non-heterosexual individuals uphold privilege, but the ability to pass as “white” is also connected to stress and invisibility (Ghabrial, 2019). Similarly, passing as heterosexual can produce its own type of minority stress (Hoskin, 2019). Bisexuality has also been reported to increase invisibility and minority stress (Ghabrial, 2019; Hoskin, 2019). In addition, old age and low socioeconomic status can exacerbate minority stress among non-heterosexual individuals (Woody, 2015; Burnes and Singh, 2016; Kum, 2017). Evidently, there exists a great diversity within different multiple-minority positions, which makes the prospect of discovering an all-encompassing understanding of multiple-minority stress slim (Follins et al., 2014; Meyer, 2015; Cyrus, 2017). Nevertheless, greater awareness of specific stressors faced by ethnic minority non-heterosexual people is needed to better address inequities and wellbeing (English et al., 2018; Almeida and Rolim Neto, 2020; Shangani et al., 2020). Despite the minority stress model's implications that worsened health is likely to follow multiple-minority status, many ethnic minority non-heterosexual individuals have been found to successfully adapt in the face of multi-layered stressors, warranting further research on resilience within this population (Bowleg et al., 2003; Follins et al., 2014). At its core, resilience is a process of stress buffering that includes anything that leads to a more positive adaptation to stress (Meyer, 2015; Vincent et al., 2020). In resilience research, the focus has predominantly been placed on the individual, yet it is critical to also recognize community resilience, where people collectively achieve and maintain wellbeing in the face of minority stress (Meyer, 2015; Kimhi, 2016; Güngör and Perdu, 2017; Lira and Morais, 2018). Positive interactions with similar-minded people and collective action can bolster resilience among sexual minorities (Difulvio, 2011; Shilo and Savaya, 2011; Bruce et al., 2015). However, involvement in non-heterosexual communities does not meditate the relationship between sexual minority stress and resilience equally for ethnic minority and white non-heterosexual individuals (McConnell et al., 2018). Separate forums therefore often become essential in facilitating resilience, and involvement in separatism is one of the most utilized and successful resilience strategies among ethnic minority non-heterosexual individuals (Lim and Hewitt, 2018; Patel, 2019; Hudson and Romanelli, 2020).

Owing to the aforementioned fact that relevant demographics and categorizations vary across countries, it is complicated to predict possible similarities and differences in ethnic minority non-heterosexual people's experiences in different geographical contexts. Additionally, both official and self-identification terminology surrounding ethnic background and sexual orientation are ever-changing. Based on the findings presented above, the author of the present study suggests that ethnic minority non-heterosexual Swedes will have unique and universal experiences in non-heterosexual spaces. From the scarce ethnic demographics that do exist, it is suggested that Afro-Swedes will be especially at risk of racial discrimination, including in non-heterosexual spaces (Brottsförebyggande rådet, 2022). From the findings regarding sexual minorities, Sweden can be viewed as an above-average progressive country in LGBTQ+ issues. Combined with the relatively small population size, wide-spread rural areas, and the divergent outlook on race/ethnicity compared to some Anglo-Saxon countries, the author expects that the findings will inform us about discrimination and resilience in a unique “periphery” of the world. As can be concluded from the multiple database searches, research on the intersection of sexual and ethnic minority positions is still lagging, and even more so in Sweden, which is why the results will be relevant in their own right.

### Aim

In line with recent recommendations regarding LGBTQ+ social psychology literature (Salvati and Koc, 2022), the present study aimed to improve diversity in the fields of social psychology and sexuality by giving voice to an under-researched group of people. The present study attempted to broaden our collective understanding of intersecting minority positions in different societal contexts by exploring ethnic minority non-heterosexual individuals' lived experiences from an array of Swedish non-heterosexual settings, guided by the following broad research question:

- What experiences do ethnic minority non-heterosexual individuals have in Swedish non-heterosexual spaces?

## Methods

### Participants

Included in the study were 13 cis-men and nine cis-women, aged 18–57 years (M = 31), with Middle Eastern, East European, South American, and North, West, and East African immigrant backgrounds. Of the 22 participants, 11 were first-generation immigrants, and 11 were second-generation immigrants. The length of living in Sweden ranged from 26 months in the country to being born here. Six of the participants were still awaiting Swedish citizenship. Nine of the participants identified as atheists, six as Muslims, five as Christians, one as Jewish, and one as spiritual. The participants' socioeconomic positions differed from unemployment to CEO. The majority of the participants lived in big cities. For the sake of the participants' anonymity, some of the demographics have been slightly changed. Instead of a specific city, the current location is presented as a small town, mid-sized city, or large city. Age is likewise not specified but instead presented as “late twenties,” “early forties,” etc., and ethnic background is presented less precisely than “usual.” These changes have been made to grant the participants anonymity, and some participants' specific wishes that the country connected to their ethnic background would not be displayed in any results.

### Inclusion criteria

Included in the present study were ethnic minority adults who self-identified as non-heterosexual, with an immigrant background that likely would increase the risk of being subjected to racism in Sweden. In other words, people who identified as non-heterosexual (including all subjective wording of sexual orientation, synonymous with “non-heterosexual”- see “self-identified sexuality” in Table 2) and who had experienced racism (i.e., had met prejudice and/or discrimination based on their ethnicity). The immigrant background was defined as foreign-born, or Swedish-born with one or both parents from another country or, as stated in the information letter: “/…/people who have a ‘non-Western' immigrant background (i.e., immigrated themselves or where one or both parents immigrated).” “Non-Western” was used to exclude people with immigrant backgrounds from, e.g., Finland (Statistics Sweden, 2022b), to preserve the focus on racism, which is more targeted against people with “Non-Western” backgrounds. No exclusion was made regarding gender or gender identity, but unfortunately, the present study did not succeed in recruiting transgender participants. The participants all identified as cis-. Therefore, cis- is not added every time, e.g., “male participants” are mentioned in the text below. Sufficient fluency in the Swedish or English language was required to participate in the present study.

**Table 2:** Demographics.

**P**	**Gender**	**Self-identified sexuality**	**Age**	**Ethnic background and generation**	**Religious affiliation/belief**	**Current location**	**Occupation**	**Interview setting + number of interviews**
1	Cis-man	Homosexual	Late forties	South America 1st generation	Atheist	Large city	Cleaner	Participant's home × 2
2	Cis-woman	Queer	Mid- twenties	Middle East 1st generation	Atheist	Large city	Student	Participant's home × 2
3	Cis-woman	Queer	Early twenties	Middle East 2nd generation	Muslim	Large city	Student	Participant's relative's home × 2
4	Cis-man	Homosexual	Late fifties	Middle East 2nd generation	Muslim	Large city	Entrepreneur	Skype × 2 + 1 meeting at participant's workplace
5	Cis-man	Homosexual	Mid- twenties	Middle East 1st generation	Muslim	Small town	Artist	WhatsApp
6	Cis-man	Homosexual	Late thirties	North Africa 2nd generation	Atheist	Large city	Photographer	Skype
7	Cis-man	Homosexual	Early twenties	Middle East 2nd generation	Atheist	Large city	Student	Skype
8	Cis-man	Bisexual	Late twenties	Middle East 2nd generation	Atheist	Large city	Scientist	Participant's home
9	Cis-man	Homosexual	Early forties	South America 2nd generation	Atheist	Large city	Actor and activist	Restaurant
10	Cis-man	Homosexual	Mid-forties	Balkans 1st generation	Christian	Large city	Event planner	Participant's office
11	Cis-man	Homosexual	Early twenties	Middle East 1st generation	Jewish	Large city	Singer and student	Participant's home
12	Cis-woman	Lesbian	Early twenties	Middle East 2nd generation	Atheist	Large city	Journalist	Skype × 2
13	Cis-woman	Lesbian	Mid- thirties	Balkans 1st generation	Christian	Large city	Chef	Hotel
14	Cis-woman	Lesbian	Early twenties	Balkans 1st generation	Muslim	Large city	Makeup artist	Participant's home
15	Cis-man	Homosexual	Mid- thirties	North Africa 1st generation	Atheist	Small town	Teacher	Skype
16	Cis-woman	Lesbian	Early thirties	West Africa 1st generation	Christian	Large city	Student	Participant's home
17	Cis-woman	Lesbian	Early thirties	East Africa 2nd generation	Christian	Large city	DJ	Skype
18	Cis-woman	Lesbian	Early forties	East Africa 1st generation	Christian	Mid-size city	CEO	Participant's office
19	Cis-man	Homosexual	Early twenties	Middle East 2nd generation	Muslim	Large city	Sales	Coffee shop
20	Cis-man	Homosexual	Early thirties	Middle East 1st generation	Muslim	Large city	Project manager	Skype
21	Cis-man	Homosexual	Mid- twenties	South America 2nd generation	Atheist	Large city	Physio-therapist	WhatsApp × 2
22	Cis-woman	Queer	Mid- twenties	Middle East 2nd generation	Spiritual	Small town	Student	Skype

### Recruitment

Over 2 years, every venue known to the author for possible recruitment was exhausted. An advertisement was carried out on the University's homepage, through numerous Swedish LGBTQ+ organizations, and on a Facebook page for Swedish non-heterosexual people with thousands of members. Snowball sampling and word-of-mouth were also utilized.

### Procedure

After initial contact was made through any of the abovementioned recruitment paths, a place and time convenient to the participant for the interview/interviews was decided upon. Most interviews took place in the participants' homes or other real-life settings of the participants' choice. Some interviews were conducted through video platforms, owing to logistic difficulties on part of the author, whose workplace was situated in Northern Sweden, on average 6 h from the participants. The geographical distances combined with financial and time constraints, unfortunately, did not allow for meeting in person with all participants. Semi-structured in-depth interviews were conducted. The interviews covered a myriad of topics, including families, workplaces, and the context of the present study, Swedish LGBTQ+ spaces. Appendix 1 provides interview questions related to the present study. In the present study, the focus is narrowed to issues concerning Swedish non-heterosexual spaces based on the fact that the participants' experiences from these were so many and so pronounced that they warranted a study of their own. On average, the interviews lasted 75 min. Some participants preferred to be interviewed in two shorter sessions instead of a longer one (see Table 2). Meeting participants more than once, be it live or *via* video link, in some instances facilitated a deeper rapport between interviewee and interviewer. At the same time, rapport was more easily established during longer interviews. Overall, rapport was not dependent on the number of meetings. However, the interview settings (see Table 2) did influence rapport. Especially the settings at “participants' homes” included a significant amount of everyday communication (not recorded or used as data) and common social “rituals,” such as having coffee together, before and/or after the formal interview. Regardless of the number of interviews and specific settings, follow-up contact was made with all participants. From the above, and maintaining participant-driven contact since the initial meeting/s, it can be safely concluded that all in all satisfactory rapport was established. The last interview was conducted right before the first wave of COVID-19 hit Sweden, which explains why the participants' experiences do not seem affected by the pandemic. Interviews were recorded, orthographically transcribed, and analyzed through thematic analysis.

### Terminology

Some terminology used in the present study might benefit from clarification. Throughout the text, “non-heterosexual” spaces/settings/world/etc., are used. However, in some instances “LGBTQ+” spaces/etc., occurs when participants themselves used this terminology or when previous research was not limited to non-heterosexuality. In Sweden, the acronym HBTQ+ (*homosexuella, bisexuella, trans- och queerpersoner*+) is used as an umbrella term, equivalent to “LGBTQ+,” in media, research, and by some of the participants in the present study. “Ethnic minority” was limited to people whose ethnic minority background increased exposure to racism in Sweden. Three of the participants had ethnic backgrounds from the Balkan region, which by some scholars might be considered “white.” However, these participants endured discrimination and racism, connected to islamophobia, and had experienced prejudice against the Roma (antiziganism) in Sweden (e.g., Granqvist, 2021). Therefore, their participation was deemed highly relevant.

### Thematic analysis

Thematic analysis (TA) was used to analyze the data. The initial coding and analysis were conducted by the author. In the second stage, all transcriptions were read and coded, and themes were reviewed and revised by a senior advisor with extended experience in methodology in collaboration with the author. TA is a widely used and easily understood method involving the recognition and analysis of meaning-bearing themes in qualitative data (Braun and Clarke, 2006; Riessman Kohler, 2008; Willig, 2013; Flick, 2014; Howitt, 2016; Clarke and Braun, 2017). The data was coded without trying to fit it into preexisting theories, while acknowledging the always-present theoretical presumptions (Braun and Clarke, 2006; Clarke and Braun, 2017). Braun and Clarke (2006) systematic procedure was used during the analysis, following the authors' six phases. In phase one, multiple readings of the transcripts and noting down initial comments were done. In phase two, the more formal coding process took place, generating preliminary codes on interesting features of the data (Braun and Clarke, 2006; Howitt, 2016). In phase three, the codes were systematically sorted to analyze how different codes formed possible overarching themes and sub-themes. Refinement of the initial themes followed in phase four, reviewing if themes should be collapsed with other themes and/or if themes needed to be broken down into sub-themes (Braun and Clarke, 2006; Howitt, 2016). In phase five, further refining and naming of the themes took place. In phase six, the final paper was produced. The analysis followed the guidelines for reflexive thematic analysis (Braun and Clarke, 2022). During the second collaborative round of analysis, there was a high level of congruence in codes and themes identified by the two coders. However, some of the initial themes were merged and all themes were refined to better capture the essence of the findings. As customary, selected quotations are embedded in the analysis to illustrate identified themes. Selected quotations of when participants spoke Swedish during the interview were translated into English, first by the author and then by the second author as well as a professional English language review agency. Quotations have in some instances been abbreviated and linguistically corrected to erase unnecessary dysfluencies and misunderstandings (Riessman Kohler, 2008).

### Ethical considerations

The data used in the present study involves special categories of personal data. However, measures have been taken to reduce identifiability to a minimum. Informed consent was obtained from all participants. Describing experiences of discrimination, mental health, etc. might create distress. However, the questions about the participants' experiences of Swedish non-heterosexual spaces followed more “neutral” issues (not addressed in the present study). The interviews ended with questions addressing possible feelings of distress experienced by the participants. Even if the participants sometimes, understandably, got emotional discussing certain issues, they assured the interviewer that they had not been offended or upset by specific questions or by the interview as a whole. This was also made sure through follow-up contact. The participants were encouraged to reach out to the interviewer if they wanted to discuss thoughts or feelings that might arise after the interview/s and were provided with contact information for available mental healthcare resources. Ethical approval was applied for and approved by the regional ethical board in Umeå, Sweden. The study was carried out following the ethical principles stated in the Declaration of Helsinki, including informed consent. Great precautions were undertaken to protect the privacy and confidentiality of the participants, and specific consideration of possible harm to the vulnerable group of interest (World Medical Association, 2013). Guidelines provided by the University's Research Ethics Committee (Mid Sweden University, 2022) were also utilized throughout the research project.

## Results

Two main themes were identified through the thematic analysis: “*Constantly contested identities*” and “*Effects and counteractions*.” These and the four sub-themes are summarized in Table 3. The analysis is structured and presented from phenomena (ingrained and intersecting ideals) to places where it unfolds (prejudiced spaces) and, lastly, to the effects (never fully human) and counteractions (representation and separatism). In other words, the themes are presented starting from a more theoretical level, moving to situated knowledge, and finally to psychological and practical implications. All results pertain to experiences within the geographical context of Sweden, except for online experiences, where it was not possible to ensure that all encountered parties had been based in Sweden.

**Table 3:** Main themes and sub-themes.

**Constantly contested identities**	**Effects and counteractions**
*Ingrained, intersecting ideals*	*Never fully human*
*Prejudiced spaces*	*Representation and separatism*

### Constantly contested identities

In this theme, experiences of the participants' identities constantly being contested and challenged, are examined. Ideals and norms surrounding ethnicity, gender, beauty, and other intersecting factors are put in the spotlight, as are spaces where prejudices and discrimination, based on the ideals, take place within the Swedish non-heterosexual world.

#### Ingrained, intersecting ideals

In this sub-theme, ideals that according to the participants dominate Swedish non-heterosexual spaces are examined. The participants first and foremost highlighted ideals related to the whiteness norm, which in turn was perceived as closely intertwined with ideals connected to religion, looks, class, and age. An intersectional identity struggle was repeatedly described:

*It's always a struggle. To be an immigrant, homosexual and attracted to older, larger men. The minority in the minority etc! It's not only heterosexual men who criticize me, homosexuals do too*. **/Participant 4**

*I have to make a choice between these two identities, and they are so intertwined. I'm either black or I'm queer and belong to that community. And you can't do anything about it, and that's pretty much how it is. There aren't many spaces where I can be both black and queer simultaneously*. **/Participant 17**

A clear majority of the participants had experienced the whiteness ideal and a lack of ethnic minority representation within the Swedish non-heterosexual world:

*Yes, when I'm in those white, queer settings I struggle to just be accepting and just flirt a little, because I can get so angry that it's so white. Not angry, but I go into this context expecting loads of queer people, and when I think of queer, I think of myself and then I don't see anyone who looks like me*. **/Participant 3**

Being black and non-heterosexual appeared to be a particularly questioned combination:

”*They get “eeeh…” (When I tell them I'm lesbian) and then they start pretending… “Oh, my God, were you out in Africa?” and I say, “Yes, I was out” and then they tell me that in Africa it is really horrible to be gay. But even here, they are also surprised, you know. /***Participant 18**

The quote above exemplifies how the Swedish progressive stance toward gay rights can have a side effect of “demonizing,” e.g., Africa as an undifferentiated homophobic continent while Swedish homophobia and racism go unnoticed (Jungar and Peltonen, 2017). Generalizing the participants' experiences, it appeared that the darker the skin color, the harder it was to also be accepted as non-heterosexual. The whiteness ideal was so pronounced that many participants felt that their own sexual identities became invisible as they were automatically identified as heterosexual. Ethnicity and sexuality were repeatedly experienced as forcibly mutually exclusive:

*One thing is that I often experience that I don't have the right to my own sexuality, rather it's a white norm that thinks “you should be hetero.” It's quite fascinating. That a dark man has to be hetero. /* **Participant 6**

Racism within the Swedish non-heterosexual context has thus far not been examined or problematized to any higher degree within media or research. This does not imply that it constitutes a minor issue for those affected. With few exceptions, participants' consensus was reached on racism being disappointingly alive and well within the Swedish non-heterosexual world, sometimes to the participants' surprise,

*So, I was friends with two gay guys who were married and they were really nice, outgoing and like, always got really dressed up and Halloween with them was so much fun, because it felt like you could just be free. But then they were, well not both of them, but one of them was pretty racist and so it was just like “right, no thanks.” And then it was just “of course two guys from the countryside aren't very (woke).” But I still think that if you are gay, or immigrant or whatever, it feels like you should be more understanding. Because you're already a bit of an outsider. Shouldn't you then try to include others?* **/Participant 22**

and, always, to their understandable disappointment:

*You know, I've noticed that the biggest racists in this society exist within the gay world, in other words: gay men. They are so awfully attached to ethnicity, your ethnic origin, how you look, if you're fit or not. I've encountered people who are like this: “No, you look like you are kind of Latino and that is not my thing.” /* **Participant 21**

Another influencing factor within the Swedish non-heterosexual world was a Muslim identity. For some participants, an actual or *assumed* Muslim identity resulted in discrimination and prejudices from white, Swedish non-heterosexual individuals:

*Yes, plenty yes, plenty of prejudices (from white homosexual men). That why I so longed to have a relationship with a Muslim. With the same background as me, practicing or not*. **/Participant 4**

The following quote describes how white non-heterosexual guests spoke Swedish when the participant had yet to learn the language and only addressed him regarding his Muslim identity:

*I remember once when I dated someone and invited his friends for dinner and served a four-course dinner to these people I had never met before, and they just spoke Swedish and I didn't understand anything during the whole evening. And then, the first thing the guy I dated said (in English) to his friends was “Did you know that* ^***^ *is a Muslim?” That was the first thing they wanted to talk with me about, and I was thinking, “Is there nothing else you can ask me?” /***Participant 20**

Even if not all the participants had experienced Islamophobia, all the participants had met expressions of overt and/or covert racism. These experiences included behaviors from white partners who had compared skin colors in an exotifying fashion or “joked” in a racist manner:

*And she was white. And so, when we would talk about politics or something or the war in Iraq, I was saying that America should not be involved, despite Iraq having a dictator, America should not be there and she said “Well, the world is over populated and some people have to die” and I was like “Why don't you die then!” Why should we die?! Because we are brown? So we broke up, because I did not want to be associated with someone that thinks like that. And she knew I was Arabic. And she claimed that she was joking, but I did not think it was a joke and I had been dealing with racism my whole life so I was done. /* **Participant 2**

One participant had a divergent opinion of what he perceived to be unfounded ‘racist shaming' within the Swedish LGBTQ+ world:

*There are million shades of white and million shades of black and million shades of Latino. It is not one color; it is a lot of things. So, this is where the problem occurs. “Oh, you would not date all blacks?!” and I'm like “No, just as much as I wouldn't date all Middle Easterners or all whites.” So yeah, there is a lot of shaming in the (LGBTQ) community, unfortunately. /* **Participant 11**

This participant meant that fingers too quickly pointed to stigmatize ideas and people as racist in Sweden. However, the participants' experiences taken together speak more of the opposite problem, with a whiteness ideal and connected racism still largely left uncriticized in Swedish non-heterosexual spaces. Many participants spontaneously emphasized how the whiteness ideal intersected with the ideals of beauty, gender, socioeconomic position, and age within Swedish LGBTQ+ spaces. Beauty is an arbitrary concept, dependent on time and culture. Still, the participants concluded that confirming present beauty ideals was an advantage in the non-heterosexual context. Ideals surrounding looks, especially the “fit body,” were more commonly emphasized by the male participants, demonstrating how gender influenced the experienced ideals:

*And everything just works and is perfect. But it gets very bare and very sexual. I believe it's because many people want to make sure things will be good in bed first. That's where the norms come in for gay men. You should be tall, you should be fit, preferably sporty and rich, and preferably light in color*. **/Participant 6**

Several of the male participants stressed (male) non-heterosexual beauty ideals, as factors with the capacity to “compensate” or worsen other dis-privileges:

*Once you're thin, it then gets, like (better). There's a “no fat, no femmes” thing, the most famous quote forever, it's almost the slogan of the gay world…* **/Participant 7**

The man below underlined the multi-faceted interconnections between ideals concerning looks, money, ethnicity, and age:

*This dream body also means “I can spend 3 h a day in the gym.” “I can afford the time.” Time is extremely precious in our society. It means that you have a good job and that you can spare the time. Top position where you can afford to work 4 h a day, then the rest (of the day) taking care of yourself. /…/ We suffer from not fitting in to the mainstream gay scene. I am a foreigner, we are poor, we are old and fat. They do not say anything like “what are you white trash doing here?” but they surely do not greet us or act welcoming*. /**Participant 1**

The strong fitness ideal that was present in the men's experiences did not have a clear counterpart among the women. An inconclusively defined ideal of masculinity was also experienced by male participants. This ideal was closely related to a sexualized ideal. The masculine ideal tended to render femininity undesirable, producing conflicting identities and insecurities for some of the male participants:

*Well, I feel that I am feminine and that I have been more feminine but that this has been broken down almost entirely. /…/ Right now, I look very masculine and then people (also) have a hard time to put the pieces together, that I'm also racialized and Muslim. They can't get those factors to fit together. People want to challenge your identity all the time and you have to learn to stay strong. /* **Participant 19**

This man underlines the associations between the ideal of whiteness and masculinity and that both femininity and masculinity were challenged. Without exception, all male participants described Swedish non-heterosexual spaces as hyper-focused on sex. Several of the men criticized this focus. Some participants gave their own explanations as to why the ideal existed:

*I mean if you give gays the rights today, like all the LGBT in the whole world. If you give them all the rights that they deserve, I think we need around 100 years until we fit back into society without being this sexualized. Because (now) they expect us to be sexualized. They expect us to be wild and crazy and if they learn that “Oh, you are in a relationship for like 12 years?”—that's a shock! /* **Participant 11**

Other participants also viewed the sexualized ideal as based on heteronormativity, where non-heterosexual people still are so “new” to socially accepted norms that it creates ideals that otherwise might not have been as prominent. Sexualized masculinity was largely dependent on whether participants were perceived as a woman or a man. A few women viewed the masculine ideal as affecting both non-heterosexual men and women, where masculinity was endorsed independent of gender. One woman pointed to what she saw as an almost mandatory “manly butch” ideal for women who recently had disclosed their non-heterosexuality. Age was another factor mentioned recurrently, even though the participants themselves were relatively young, and was often seen as connected with the aforementioned ideals:

*There is an age fixation, absolutely. I think that it is related to the muscular fixation. It is very superficial, just at the surface level. Yeah, everyone wants to be with someone who is young and fit…* /**Participant 9**

Some participants, both men and women, worried about their own aging and the perceived lack of senior forums for non-heterosexual people. Regardless of the ideal discussed, many participants stressed that these ideals were mere reflections of the mainstream world:

*Well, what I experience as the norm is, like, or what I see most of, just like with everything else, is like, thin, white people. Like, good-looking people. And that's the norm in the LGBTQ world too. Yes, well the first thing that comes to mind is like a standard Swedish LGBTQ person, who is like, an androgenous, thin, mega-gorgeous Ruby Rose-like person (laughs). /* **Participant 12**

*Very many people have always been very clear that they are not interested in Asians, they aren't interested in fat people who don't have a six-pack, and all those norms exist everywhere, and there can't be any difference there between heterosexuals and homosexuals. /* **Participant 10**

Being white, rich, good-looking, and not “too” old provides privileges.

#### Prejudiced spaces

In this second sub-theme, the participants' situated knowledge of the ideals, in different non-heterosexual spaces, are outlined. The aforementioned ideals were viewed as enacted on interpersonal as well as institutional levels, offline, and online. In Swedish non-heterosexual club and party scenes, prejudices were experienced by the participants. When asked about how white non-heterosexual women act at parties/clubs, the female participant described it as follows:

*A bit weird, once they relax and they've had a bit of alcohol, it gets a bit easier, they don't think so much “she's a foreigner, she's a bad person,” but at the same time many think that people from Bulgaria are dodgy. That's the typical picture, that we are people who steal and lie and so on. But what I say is that you can't tar everybody with the same brush. /* **Participant 13**

Swedish Pride festivals, especially Stockholm Pride, were similarly described as steeped in the abovementioned ideals and participants questioned who Pride is catering to:

*During Pride they try to include as many as possible but there exists a white cis-normativity within the gay world, where it is very clear that white cis-gay men with a higher income dominate. People, who can afford to travel a lot, buy a lot of clothes or things, and they constitute a large difference from the rest of the LGBTQ*+ *world, where there exist many poor queers. /***Participant 9**

Several participants viewed Pride, in addition to being “white,” as an event that over time had become inaccessible for the socially and economically vulnerable. In addition, some participants saw Pride as overly focused on sex, with BDSM workshops and similar happenings, which did not feel representative for them. On a more institutional level, participants discussed the best known, and for many laymen, the *only* known LGBTQ+ organization in Sweden: The Swedish Federation for Lesbian, Gay, Bisexual, Transgender, Queer, and Intersex Rights/*Riksförbundet för homosexuellas, bisexuellas, transpersoners, queeras och intersexpersoners rättigheter* (RFSL). This organization is a non-profit association that receives state-funding and acts as a highly influential voice within the Swedish non-heterosexual world. All participants knew of RFSL and a majority had experiences with the organization. Some of these were very positive:

*Yes, I love it. I am actually going there today. And on Fridays there are a lot of refugees that come, it is like a special evening and you cannot share who was there, it is supposed to be a safe space*. /**Participant 2**

In general, RFSL had a better reputation among participants who had been in Sweden for a shorter period, while second-generation immigrants usually were more critical of the organization. Even among the participants who were positive regarding RFSL's work, there existed doubts concerning the organization's focus and willingness to take action:

*Yeah, it feels kind of like home. But still RFSL feels really, really weak. It does not support, like well I have two transgender friends and they are still in camps. Like RFSL should be there and speak for them. The thing is that RFSL only care about the Pride festival and parties; it is superficial. But it is hard because they cannot go too far. They do not want to lose their privileges as an organization. /* **Participant 5**

Several of the participants had experienced RFSL as rather unapproachable or unwelcoming. Examples ranged from RFSL employees and members who continued to speak Swedish even when people that didn't know Swedish were present to diversity and representation within the organization. The woman below describes previously working at RFSL and, later, being asked to help the organization with an anti-racist plan:

*I've worked at RFSL and it was really bad. All their racialized activists work for free and RFSL youth section is sooo white. There was a congress 2 years ago and I was like “You don't even have one racialized person in your board.” And someone said, “Well, it is hard to find them.” I'm like “Them?” “Find them?” and said “That means that your organization is so unwelcoming that people don't even want to come there.” And now RFSL want some anti-racist plan of action and ask, “Hi, could any of you come here and write our anti-racist plan of action.” And, of course for free “There is no emolument because we are short of money, but we will offer you coffee.” And I'm like, “You have among the highest salaries ever,” because they have crazy high salaries, “and you can't even pay for people to do your fucking plans of action, that you don't even follow.” I was like “You should be ashamed of yourselves” and they were just “It's voluntary.” Yes it is, and I hope no one helps you with it! /* **Participant 16**

Concern was voiced regarding the discrepancy between the organization's official anti-racism marketing and the fact that the vast majority of positions of power within RFSL were earmarked white:

*But now I work at a bar here in xx (area of city), and I'm also chairman in RFSL xx (name of city), so I actively work with LGBTQ*+ *questions. For example, we work with “sex experts” (sexperterna) and HIV prevention and different sexually transmitted diseases /.../. Still, they always have, like, one racialized person in the RFSL's board, kind of. They have one, not two, you know. I'm the only racialized chairman in this city. RFSL have very few chairmen who are racialized. /***Participant 9**

Information and resources connected to RFSL and many other Swedish non-heterosexual spaces can at present be found both offline and online. One of the most frequently discussed online spaces was online dating sites and apps. Gender was here, again, an informing factor in experiences of prejudices and discrimination, usually to the detriment of the male participants. For many of the men, the Swedish non-heterosexual online dating scene was experienced as the most blatant arena for discrimination and dehumanization:

*On Grindr it is sick. Like “I want to suck your Arab cock,” or just in general reducing me to an object. Like I'm not a human, you know. Then when you answer “No thanks” they become angry and call me “Blatte*^2^ *jävel” and say “Go back to your country,” and then you get to see the real person. /***Participant 19**

Participants viewed white non-heterosexuals problematic actions as aided by the under-problematized design of many of the dating platforms:

*I think it (racism) is really tedious because it exists and everyone knows about it, but nothing is done to prevent it. It is just allowed, and on communities like QX and Grindr (dating sites/apps), the platforms still provide info boxes to state your ethnic origin, what (ethnicities) you like and all that stuff. As long that this still exists, I believe that the racist culture will also continue and that is something I think is really important to discuss. /* **Participant 21**

Several of the male participants were baffled by how white non-heterosexual Swedish men approached them in online settings:

*I notice that when Swedish people (men) talk to each other or the first thing that they will write to me online is “Horny?” For me that is not normal, I am always like “Hello, how are you?” I do not think we do that (sexual salutation) back home. So Swedish people think it is ok to come to me out of the blue and ask if I am horny. I do not like that*. /**Participant 15**

The sexualized approaches online were viewed as intertwined with existing ideals of fitness and whiteness. Some of the female participants had also noted ideals connected to whiteness and beauty in online dating, but for the women, the most discussed experience was one of scarcity:

*I've tried online dating and there are so many more options if you are a gay guy. I know a gay guy and he's just like “It is as easy as ever, wherever you are in the world you can just go in on Grindr.” And I'm like “Okie dokie, well it's not the same here… for us, on that front” (Giggle), I've used Tinder and it kind of sucks after 25 min. There isn't a lot there. Like, you swipe three times and then there are no more women. /* **Participant 22**

This woman lived in a small town and explained that, even when she expanded her geographical search range to include all of Sweden, there were still extremely few non-heterosexual women to be found. One woman, who self-identified as butch, had experienced exclusion in Swedish online dating because other women misgendered her:

*Many times, women on lesbian sites have written: “This place is only for girls!” to me*. /**Participant 13**

While many of the women struggled to meet other women online, two of the male participants had taken the opportunity to conduct experiments online. The following quote describes a homemade experiment that examined the role of ethnicity and response rate:

*A friend and I tried Grindr in Gothenburg and we set up two almost identical profiles, where all the information was the same but with different photos. One profile showed a white person and the other a person from the Middle East, and both looked pretty good. We directly saw who got most answers… Sure there are some problems with this (experiment), but overall, well, the white profile got more answers. /* **Participant 7**

Another man had looked closer at sexualized ideals and actions in the Swedish non-heterosexual (male) online scene, through the following dick pic experiment:

*I set up a fake profile with a photo, where you really didn't see me at all, and sent this photo of only my genitals to see how many actually were enthusiastic about that. To see, well, who would take that as something positive and continue the contact and like “Yes, should we meet?” And there were surprisingly many who did that, and then there were those who “Uhm, I would like to see a face too, I want to know who I'm talking to” and then there were some who thought it was totally idiotic to send a dic pic without having had any prior conversation. But there were a good deal (who liked it)! /* **Participant 21**

With the above amateur experiments, the analysis will move to the effects of the ideals experienced in different non-heterosexual spaces and further elaborate on what a multiple-minority status entails within the Swedish LGBTQ+ world at present.

### Effects and counteractions

In this theme, the effects of upholding an ethnic minority status in the Swedish non-heterosexual world are addressed. This theme highlights experiences of alienation, exotification, tokenism, and the connected “outsider,” not fully human, feeling. Difficulties in dating and expectations of adaption in relationships with white non-heterosexual individuals are also discussed. The two most commonly utilized counteractions against the experienced difficulties were increased representation and participation in communities favoring separatism.

#### Never fully human

This sub-theme focuses on some of the effects of the hindering ideals and discrimination within Swedish non-heterosexual contexts. These effects include feelings of alienation, dis-belonging, sadness, and, basically a sense of not being accepted as fully human. Two words could be said to summarize the experienced effects of the findings discussed in the themes above, namely, “exotified” and “outsider.” The outsider feeling was often described as always present:

*Yes, I always felt like an outsider, wherever you are from, from a church, I am an outsider, if you mean the gay community, I am an outsider. /* **Participant 11**

Participants recurrently discussed their experiences in the Swedish non-heterosexual world using “outsider” or equivalent words. Frequently, white non-heterosexual individuals made unwanted positive and/or negative remarks about presumed ethnicity-based characteristics:

*When they ask me “Where are you from?” and I answer they can say nice things, like I would feel better in my identity then: “Oh, how nice, xxx (participant's ethnicity) guys are hot,” well, those kinds of really cheesy, meaningless things that they maybe think are nice for me to hear, so I'll be happy about my ethnic identity, I don't know! It sometimes feels like people say things because in reality they think the exact opposite! (Laughter). In my experience, being a person with another background can be really interesting for some people and that's also a form of racism, or some kind of exotifying view of, like an object. /* **Participant 20**

Even though many participants presented a light-hearted and humorous account of their experiences within the Swedish non-heterosexual world, it is safe to conclude that a constant reminder of not belonging takes a toll on the recipient's identity and wellbeing. Exotification was a common experience, and attention and praise based on exotification often left an unpleasant aftertaste:

*During my 1st years here, I was out a lot and met a lot of men. There were very many white men and I couldn't really understand why they liked me so much, but that's when I got introduced to exotification and sexualization of my ethnicity. I thought of it as a positive thing, because I wasn't very educated. I didn't understand how tragic it was that I felt exotic. /* **Participant 19**

The woman below had initially had an optimistic outlook on relationships between ethnic minorities and white non-heterosexual persons, but based on her friends and own experiences, she later reached a more pessimistic conclusion:

*I have many racialized, queer friends and I realize that they could go out with white women, but when I ask them they say that they couldn't because, because it is so anti-racist. And I feel that it's a pity for them because they would enjoy being with this person. But I myself have personal experience, based on the fact that romantic relationships are so private, personal, and so intimate. I don't want to be exotified by my own partner. Not everything is political, but everything is nonetheless political*. **/Participant 3**

Close to exotification is the sexualization of non-white ethnicities. This was a gender-dependent factor and a recurrent experience specifically among the male participants. Exotified sexualization involved expectations, or demands, based on colonial ideas about the non-white body as overly sexual and tailored for white exploitation:

*I think there is this classic view that you are expected to be a sex athlete and there is a really strong connection to sex. Like you are expected to always want (to have sex) and they can't understand when you don't want them*. /**Participant 6**

Connected to this were experiences of white Swedish non-heterosexual men who wanted to have sex with men of other ethnic backgrounds, but only sex:

*But there are people who want to travel and have sex with people from different nationalities, but at the same time they want to marry a peer, someone who is similar and have the same values and preferably are successful, they want to meet a white middle class man. /* **Participant 1**

In the quote above, the intersections of whiteness and class ideals are highlighted. Dating white non-heterosexual individuals is of course not a given goal, and indeed, some participants stressed that they did not want to date white non-heterosexual individuals. This standpoint was partly based on what they viewed as a preoccupation with superficial class markers among white non-heterosexual people:

*So yeah, in that way I wouldn't date (Swedish boys) like, maybe, it might happen, but for now yeah, everyone is so concerned about the social status you know, what you do for work, and where you live. How you look, where you buy your clothes, and all these things, and for me it is kind of, like shallow, you know. /* **Participant 11**

However, a high socioeconomic position was not necessarily something that improved dating opportunities within the Swedish non-heterosexual world. The woman below worked as a successful CEO but suspected that her ethnic background remained the overshadowing reason for her dating difficulties:

*It is impossible! (laughter) Not only hard, but impossible. It is impossible. I don't know if it's because I'm black or a colored person, a person of color, I don't understand. /.../ Here it is a problem, even when you're in a relationship it is a problem. Culture is a reason used. You know “we don't understand each other, because you're from a different culture.” But I don't understand! I don't know if it is because of the language, but even those who know Swedish have failed marriages. /* **Participant 18**

For several of the women, expectations of adaption, speed of relationship disclosure, and lack of cultural understanding were highlighted as stressful, recurrent features in dating white women:

*When I've dated Swedish girls, they have very soon wanted to meet my family and I haven't been comfortable with that, but I've taken them home anyway. But then it's lucky that I can speak two languages and can say “a female friend” to my mother in xx (their language), and to the girl I say in Swedish “this is my mother” and she thinks that they've been introduced. Because, I just don't have the strength... /* **Participant 14**

*Yes, well I feel like it would probably be difficult in that, I think that maybe there would be expectations on how you are supposed to live, or how open you should be and that's not really the understanding, that's what I've always felt quite early on when I've dated white women. The understanding doesn't exist at all*. **/Participant 17**

Reconnecting to the first theme, the beauty ideals had additional negative effects, especially on male participants. The man below reflected on the challenge of creating a healthy relationship with fitness:

*I ended up in an unhealthy relationship with a very fit guy and I wasn't fit myself at that time and then I felt such a pressure to work out. Since that day, I've tried to convert exercise into something that is good for me, instead of a compulsion. I don't know if I can be totally neutral though, because I'm also affected by the norm and this (fitness) ideal is a part of it. It is so fixated on looks, and you can go far in this (LGBTQ) world if you look good. You can get almost anything you want and a lot of friends. /* **Participant 19**

Another man felt that fat phobia within the Swedish non-heterosexual community affected his self-image and his relationship with his partner:

*Sometimes I feel how the prejudices (surrounding obesity) affect me and then I tell my boyfriend to work out and say nasty things to him. /* **Participant 4**

Lastly, both men and women highlighted tokenism, where they involuntary became token figures, as a tiresome experience within Swedish non-heterosexual spaces:

*After a while I became tired, like “I can't take being the token queer African girl anymore” who is expected to just stand there and “Look at me, I'm queer, I'm black.” I became so tired of it and thought, “Shit, I need to look after my family (wife and children) and ensure that we all have the energy to, like, continue to live.” Because just living is a struggle… /* **Participant 17**

*But what we feel is that we've been a bit of the token figure in different forums when we... we get invited e.g., to speak at Pride about racialized queers. And you're often alone or one of very few racialized queers sitting talking about what being a racialized queer is like. And we're collectively all tired of being the token figure that gets invited. /* **Participant 9**

The participants' reasoning is in line with previous findings where tokenism has been described as a particular version of exotification, turning ethnic minorities into accessories in otherwise predominately white spaces (Kehl, 2019). Merging the experiences of exotification, sexualization, rejection, and tokenism, the distinctive psychological effect of being an ethnic minority non-heterosexual within the Swedish non-heterosexual contexts can be summed up as “never fully human,” with corresponding difficulties, including minority stress symptoms. The man below described the collective experience of ethnic minorities within the Swedish non-heterosexual world as one of the accessories and animals:

*The white gays have their thing, they have their grand parties and pride and all that jazz, but it is obvious that the racialized gays only are, this feels crass to say, but almost like animals. Just an accessory. I think that you are placed in an unhealthy quota. /* **Participant 19**

In the next sub-theme, the different ways used to cope with the abovementioned experiences are examined.

#### Representation and separatism

This last sub-theme focuses on the phenomena that the participants highlighted as effective in combating experiences of alienation, exotification, and discrimination within the Swedish non-heterosexual world. Representation and separatism were viewed as vital in many ways. First, a recap on why the increased representation of ethnic minority non-heterosexual people in Sweden is needed:

*Until very recently, when you saw LGBT people in the media and stuff, you saw just one kind of LGBT person; usually white, homosexual men for example, and the rest of the LGBTQ community was not as well-represented, to that extent. /* **Participant 17**

The participants viewed all outlets that improved the representation of the under-represented in Swedish non-heterosexual spaces as extremely valuable. Below, praise is given to a summer camp for non-heterosexual women, arranged by a non-profit organization (“Lesbian power”), where diversity was implemented:

*Yes, they're very good at that. A lot of people apply and then they pretty much work with quotas, so that it's about half-half or so, and there are a lot of different kinds of people and different gender identities and backgrounds and experiences and ages and everything, so they've really given it a lot of thought. /* **Participant 12**

Other participants had noticed positive changes in representation online, which helped them feel less isolated in their multiple-minority identity:

(Regarding Instagram) *Yes, God, loads! Now that I've admitted to myself that “OK, I am gay” well then there's been more and more. And now I've become like… that... “Well there are so many” and, you know, people in exactly the same situation (as me), because, you know, you always think that you're alone in your situation or alone on the planet. So there are many, very many. Which I think is really great, that there are people to look up to. Like... “I want to achieve that too.” /***Participant 22**

Some participants had in various ways tried to actively broaden representation within non-heterosexual settings. One participant had a YouTube channel, where Swedish non-heterosexual women with a variety of ethnic backgrounds discussed sexuality-related issues. Swedish state TV had contacted her about bringing her show to TV, but she declined:

*We received sponsor offers, but then we weren't allowed to say certain things, and SVT (Swedish state TV) was interested, but then we couldn't use certain faces because they weren't “right” for TV. So I preferred to carry on alone. /* **Participant 14**

Ideals surrounding looks intersected with the participant and her YouTube panel members' experiences, and Swedish TV missed out on a chance to promote a wider representation of non-heterosexual people to their many viewers. Another form of representation strategy was used by participants who chose to live “normal” lives, in contrast to ideals that they perceived as dominant:

*I have fought for a long time to have that feeling, that I am normal. Not to follow the straight norm, don't get me wrong, but that I am normal. I am a regular human being you know, so because I am gay I am not gonna go (as Swedish gays do) topless on drugs in Belgium, going crazy and having sex with… You know what I mean*. /**Participant 11**

Another discussed issue, connected to representation, was the “goodness” image of non-heterosexual people. In the quote below, a man describes how he in his work attempts to nuance the image of LGBTQ+ people:

*It's pretty much like I usually say when I visit schools, you have to be aware that just because we are fighting for the rights of LGBTQ people, it doesn't mean that all LGBTQ people are good people. There are gay men who are Nazis and there are gay men and lesbians who are fascists and racist and murderers and abuse their partners and the rest of it. Just because we are fighting to get the same rights as heterosexuals, it doesn't make us good people, and that's something that, well, there are many people who don't consider that. It's like always goodness. /* **Participant 10**

The most common tactic to overcome, or survive, existing discrimination in Swedish non-heterosexual settings was demonstrated in separatism. A majority of the participants had accomplished a sense of belonging through the formation and/or involvement in double-separatist forums. Trying to find shared experiences was a common goal, and separatism was experienced as an effective way to cope with multiple minority statuses:

*And then one of us who was openly queer wrote in the bigger group: “We have started an LGBT group now, if you feel like this applies to you, then join the smaller group” and within a week we had like a 100 members and that was like, these are really small figures in a bigger scale, but for us, as blacks in Sweden, to know that there is a 100 of us was like “what, that is crazy!” (Laugher). So that was a real eye-opener and it was amazing! /.../ These separatist forums simply allow you to find people with the same experiences as you, in this duality that we live. /* **Participant 17**

I think we blattar^*^ *are starting to get much smarter (we know), that we can't trust white boards of directors or organizations, that if we want to do something we have to do it ourselves. We reach out our hand to each other. Reach it out, get it slapped down, next try. /***Participant 19**

Many of the participants had left “mainstream” Swedish non-heterosexual spaces in favor of the separatist rooms, where both their sexual and ethnic identities were embraced. Several participants reflected on how they initially had questioned whether separatism was needed. The conclusion was always yes. The separatist forums acted as arenas for both serious political discussions and more undemanding activities:

*In our separatistic forum we meet people who share the same experiences. There has been a lot of talk about the stress of finding a life here (in Sweden) but also just hanging out and drinking coffee and listing to Middle Eastern music and things like that. And we also organized a lecture by a gay imam from France. /* **Participant 20**

Some of the participants had encountered criticism regarding the separatist forums from non-heterosexuals who felt excluded and elaborated on why this criticism was unfounded:

*Yes, mostly from white people, who get upset that they can't be part of it. But I don't see it as a problem, or rather I don't think we need to worry about that criticism, because it's like this, there are thousands of other places for everybody else to be part of. And, you know, we need these kinds of breathing spaces. So, I don't really see anything bad about it. /* **Participant 12**

As evident from this theme, several participants actively worked on creating inclusive spaces for ethnic minority non-heterosexual people and improving representation through blogs, social media, participation in panels, and giving interviews, including those that made the present study possible.

## Discussion

There remains substantial work to be done to further our understanding of how intersecting identity positions, and systems of inequality, impact people's lives in different contexts (Nichols and Stahl, 2019). The present study aimed at providing a small contribution to our combined knowledge of multiple-minorities' experiences, from the viewpoint of ethnic minorities' experiences of non-heterosexual spaces in Sweden. The results largely endorse previous international findings on a multitude of problematic and intersecting ideals, with negative consequences for those who do not fit the ideals, within non-heterosexual spaces (e.g., Farmer and Byrd, 2015; Tang, 2017; Grollman, 2018). A clear majority of the participants felt alienated from many different Swedish non-heterosexual spaces. The present study's recruitment process, where, unexpectedly few—based on the high percentage of people with a foreign-born background in Sweden—participants were found through established Swedish LGBTQ+ channels, confirms the participants' experiences of marginalization and invisibility. From the testimonies in the present study, a majority of Swedish non-heterosexual spaces present obstacles to positive belonging for ethnic minority non-heterosexual people.

Unbeknown to the author until after completion of the present study, the sub-theme “never fully human” corresponds very well with documented lived experiences by a Canadian researcher with an African Caribbean background who has analyzed how her ethnic background resulted in feelings of dis-belonging and “never seen as fully human” in the academic world (Henry, 2015). Again, it is important to underline that the problems revealed in the Swedish non-heterosexual world are not isolated from other arenas of life or other geographical areas. The participants' testimonies are in line with previous findings on ethnic minorities' struggles with the whiteness ideal racism in predominantly white non-heterosexual arenas (e.g., Cyrus, 2017; Ghabrial, 2017; Lim and Hewitt, 2018; Kehl, 2019; Patel, 2019), and their experiences of racism and exotification online echoes previous findings on Nordic LGBTQ+ online dating sites and apps (Shield, 2016; Svensson, 2016; Miller, 2019). Internationally, many dating apps for non-heterosexual men are known for being sexualizing in their nature (Gudelunas, 2012; Hall et al., 2012; Miller, 2015; Tziallas, 2015; Parisi and Comunello, 2016), an experience shared by the male participants in the present study. The scarcity of online dating opportunities, experienced by the female participants also corresponds with previous research (e.g., Duguay, 2019).

Islamophobia was viewed by some of the present study's participants as connected to the whiteness ideal, a link found in previous findings as well (e.g., Abraham, 2009). However, only six of the participants identified as Muslim, and the majority of them were “non-practicing,” so no major conclusions can be drawn regarding what impact religious affiliation has on experiences within Swedish non-heterosexual spaces.

Previous research demonstrated that navigating multiple sources of discrimination negatively affects psychological wellbeing (e.g., Ghabrial, 2017; Schmitz et al., 2019). The present study examined effects that the participants specifically connected to Swedish non-heterosexual spaces and does not claim to comprehensively analyze factors affecting the wellbeing of ethnic minority non-heterosexual people in Sweden. However, it is postulated that repeated discriminatory experiences within any context have a significant negative effect on a person's wellbeing. From the present study's result, and previous research on intersectional minority stress theory (e.g., Velez et al., 2015; Cyrus, 2017; Schmitz et al., 2019), it is concluded that existing ideals and practices within a multitude of Swedish non-heterosexual settings likely increase multiple-minority stress in ethnic minority non-heterosexual individuals in Sweden. The general lack of representation contributes to identity conflict revolving around difficulties to be a non-heterosexual and ethnic minority at the same time within white non-heterosexual spaces (Ghabrial, 2017; Kum, 2017; Chiang et al., 2019; Kehl, 2019)—findings that the combined testimonies in the present study substantiate. No single identity category can satisfactorily explain a person's experiences in any context (Magnusson, 2011; Geist et al., 2017), and drawing strict boundaries between one aspect of “otherness” and another would diminish the participants' truly intersectional experiences. Different positions and identities can act both aggravating and “compensatory,” e.g., good looks can “override” some of the ingrained whiteness ideal and discrimination in Swedish non-heterosexual spaces.

One important issue when attempting to apply an intersectional framework concerns similarities in experiences across categories commonly viewed as very different (Cole, 2009). In the present study, one might have expected to find greater differences in experiences based on first- vs. second-generation immigrant status. However, regardless of the length of living in Sweden, the similarities in experiences within non-heterosexual settings were far more pronounced than the differences. Gender differences were however found, especially concerning ideals connected to fitness and sexualization, which the male participants were more subjected to, which is in line with previous findings (e.g., Schmitz et al., 2019).

Previous research demonstrated that stressors experienced by ethnic minority non-heterosexual people can be heightened with age, and low economic social status (Burnes and Singh, 2016; Velez et al., 2019). As the sample in the present study was relatively young, few lived (not speculated) conclusions on age as an intersecting factor can be drawn. The few participants that were middle-aged or older, however, highlighted aging as an additional hindering stressor within non-heterosexual spaces. Regarding class, with a few exceptions, all participants were middle class, making the specific impact of different socio-economic positions difficult to explore thoroughly. The same was true concerning how rural vs. urban living affects ethnic minority non-heterosexual people, as the overwhelming majority lived in large cities.

A counteraction to the negative experiences in Swedish non-heterosexual spaces was found in the formation of, and participation in, separatist forums. The present study proposes that participation in such forums is a significant resilience mechanism used to mitigate multiple-minority stress and dis-belonging. This suggestion is in line with previous findings on how separatist groups function as a stress-buffering and successful coping tool, by providing spaces for acceptance, support, and collective action for ethnic minority non-heterosexual individuals (Lim and Hewitt, 2018; Patel, 2019; Hudson and Romanelli, 2020). Thus, the present study's results validate previous findings on the importance of community resilience (Meyer, 2015; Kimhi, 2016; Lira and Morais, 2018). Resilience demonstrated through participation in separatist spaces should be encouraged through sufficient state funding of these forums. The positive effects of separatism do however not negate the source of the problem, which is discrimination within “mainstream” Swedish non-heterosexual spaces.

It remains critical to question whom Swedish LGBTQ+ organizations represent. Some of the assumptions in the present study are in line with previous findings, which have highlighted that even when white LGBTQ+ organizations claim to be antiracist they nevertheless uphold discriminatory power dynamics and exclude ethnic minority non-heterosexual individuals from leadership positions (Han, 2007; Woody, 2015; Lim and Hewitt, 2018). Greater diversity and representation within the Swedish non-heterosexual world is, in conclusion, warranted. The Swedish healthcare system also needs improved knowledge about the specific struggles affecting ethnic minority non-heterosexual individuals to adequately be able to provide support, if needed (Holley et al., 2019).

### Strengths and limitations

The present study constitutes a snapshot from an under-researched group within an under-scrutinized non-heterosexual context. Situated in Sweden, one strength of the present study is that it advances the field of minority psychology through important insight into how multiple minorities are treated in non-heterosexual spaces in a country often viewed as leading in human rights and equality. Another strength is that the study moves beyond ethnic sub-groups by demonstrating strikingly similar experiences independent of specific ethnic minority backgrounds and length of living in Sweden. The intersectional lens utilized highlighted how ideals beyond the whiteness ideal influenced the participants' experiences in non-heterosexual spaces. Perhaps most pronounced as intersecting factors of importance were ideals of beauty, particularly for the male participants, who also experienced sexualization to a high degree. Though conceptionally difficult to define, ideals surrounding the body (e.g., fitness) and beauty are concluded to be important factors to include in intersectional work. One limitation in the present study is that some of the concepts used might not readily be applied in other geographical contexts, as the meaning and categorization of, e.g., “ethnic minority” and “non-heterosexuality” vary among and within countries. Central factors in intersectional understanding, such as age and class, could not be sufficiently explored in the present study owing to the homogeneity in the (small) sample, which is a limitation. Another limitation concerns the generalizability of the findings. People who fall outside the utilized recruitment paths, and who perhaps also are the most vulnerable among ethnic minority non-heterosexual people in Sweden, are largely still not heard. This includes closeted, rural-living, and/or elderly ethnic minority non-heterosexual individuals. Also, there are several additional affecting factors, e.g., gender expansive identities and (dis)ability that are not addressed in the present study.

### Positionality

The author of the present study identifies as a cis-gendered, middle-aged, Swedish-North American, white lesbian, with a conservative Christian background. The senior advisor who assisted in the analysis identifies as a Swedish cis-gendered, heterosexual professor. Having only partial shared identity experience with the participants was found to be beneficial during the interviews, as the author could not take anything “for granted” based on lived knowledge. Instead, the participants were prompted to describe their unique experiences. The same was true in the analysis.

### Future directions

Several of the findings in the present study would benefit from further examination. International comparative studies could be especially valuable concerning discrimination, resilience, and wellbeing among ethnic minority non-heterosexual people. It seems plausible that the situation for ethnic minority non-heterosexual individuals in less liberal countries is similar or worse compared to Swedish findings. The extent of such differences is important to explore. Further research is also promptly needed to explore the effects on ethnic minority LGBTQ+ people of the hostile backlashes that co-occur with recent advances in LGBTQ+ rights in many European countries (ILGA—Europe, 2022; Salvati and Koc, 2022). Longitudinal studies to see if ethnic minority non-heterosexual individuals' attitudes toward mainstream non-heterosexual spaces change with time spent in Sweden (or other countries), as well as experimental studies on intersecting ideals within the non-heterosexual world would contribute to our understanding of privilege and belonging. As the probability of the LGBTQ+ world rapidly changing ingrained ideals and power distribution seems remote (Woody, 2015; Lim and Hewitt, 2018), individual and community resilience among ethnic non-heterosexual people within this world needs to be better understood. The burden of discrimination should however not be placed on the discriminated, and e.g., state-funded LGBTQ+ organizations should receive incentives to change into truly including spaces that promote belonging for all sexual minorities.

## Data availability statement

The raw data supporting the conclusions of this article will be made available by the authors, without undue reservation.

## Ethics statement

The studies involving human participants were reviewed and approved by Regionala Etikprövningsnämnden i Umeå (Regional Ethics Committee in Umeå). The patients/participants provided their written informed consent to participate in this study.

## Author contributions

EM formulated the research question, did the data collection, executed the analysis, and wrote the paper.

## Footnotes

^1^Same-sex marriage was legalized in Sweden in May 2009 (Statistics Sweden, 2022a).

^2^Originally a racist slur, “blatte” was reclaimed and used as a self-identifier by several of the participants. To the author's knowledge no equivalent expression exists in English.

## Appendix 1: Interview guide—Ethnic minority experiences within the Swedish LGBTQ+ world

What do you think of when I say “The LGBTQ+ community?”

Do you feel welcome and integrated with the LGBTQ+ movement?

Do you feel at home in the LGBTQ+ community?

Do you have LGBTQ+ friends?

Do you participate in Pride, and if so; how have those experiences been?

Are you active in RFSL (National LGBTQ+ organization)?

Do you use dating sites or apps, and if so; what are your experiences?

What's your experience with LGBTQ+ clubs?

Have you encountered racism in any LGBTQ+ setting and if so; how has it been demonstrated?

Is there something that I haven't addressed that you find important to your experience in different Swedish LGBTQ+ spaces?
